# Associations Among Self-rated Oral Health, Subjective Oral Conditions, Oral Health Behaviours, and Oral Health-related Quality of Life (OHRQoL)

**DOI:** 10.3290/j.ohpd.b3858591

**Published:** 2023-02-02

**Authors:** Nan Yin, Wenhao Li, Haoyun Zhou, Yanyong Zhang, Wei Zhang, Wei Ding, Hui Ge, Shunhua Zhang, Shengkai Liao

**Affiliations:** a Graduate Student, School of Stomatology, Bengbu Medical College, Bengbu, Anhui, China. Collected the data, conducted the study, performed the statistical analysis, drafted the initial manuscript.; b Graduate Student, School of Stomatology, Bengbu Medical College, Bengbu, Anhui, China. Collected the data, conducted the study.; c Professor, School of Medical Imaging, Bengbu Medical College, Bengbu, Anhui, China. Made critical revisions of the manuscript, supervised the study, contributed substantially to study concept and design.; d Professor, Department of Stomatology, First Affiliated Hospital of Bengbu Medical College, Anhui, China. Contributed substantially to study concept and design.

**Keywords:** oral health behaviours, oral health-related quality of life, self-rated oral health, structural equation modeling, subjective oral conditions

## Abstract

**Purpose:**

To evaluate the relationship between self-rated oral health, subjective oral conditions, oral health behaviours, and oral health-related quality of life (OHRQoL) in Chinese college students.

**Materials and Methods:**

An online cross-sectional survey was conducted, inviting college students from eastern China to participate. A total of 1708 participants were included. A structural equation model was constructed to explain and assess the associations among self-rated oral health, subjective oral conditions, oral health behaviours, and OHRQoL.

**Results:**

Self-rated oral health had a direct positive effect on subjective oral conditions and OHRQoL. Oral health behaviours had direct negative impacts on subjective oral conditions and OHRQoL as well as on tooth condition perception and oral health interventions. Subjective oral conditions had a direct positive effect on OHRQoL. There was a positive correlation between oral health behaviours and self-rated oral health. In addition, subjective oral conditions partially mediated both the effect of oral health behaviours on OHRQoL and the effect of self-rated oral health on OHRQoL.

**Conclusion:**

There were influential associations between self-rated oral health, subjective oral conditions, oral health behaviours, and OHRQoL among college students in eastern China. Making the most of their association can be a guide to radically improving the oral health of college students.

Oral health is one of the major components of overall health, and improving oral health plays a significant role in enhancing the population’s overall quality of life.^13^ Compared to others, college students represent a highly educated population that is more likely to be educated about oral prevention and care and to change poor oral habits.^14^

The oral health-related quality of life (OHRQoL) as a multidimensional construct is often used in oral health research to measure the impact of oral disease and oral conditions on patients’ general condition and daily behaviour.^6,19^ Measuring a student’s OHRQoL is important for conducting oral health surveys and oral disease prevention, as well as for determining the effectiveness of clinical treatment of oral diseases.^13^

Epidemiological studies often use self-rated oral health as an indicator of a subjective experience that has an important influence on people’s well-being and quality of life.^7,23,26^ Self-rated oral health is one of the most direct responses of patients, reflecting the individual’s subjective experience of physical and mental health, clinical symptoms and adverse conditions.^1,18^ A previous study showed that the monitoring of young people’s oral health should also include information on testing self-rated oral health.^11^

The main factors associated with self-rated oral health include clinical examination of oral factors as well as subjective oral factors.^18^ Subjective oral conditions are primarily derived from individual judgments, that is, an individual’s subjective perception of oral conditions.^16^ Currently, subjective oral conditions are used more often in dental care to determine the severity of a patient’s disease rather than the conventional, solely objective oral conditions indicators.^24^

Poor oral health behaviours may adversely affect OHRQoL.^2^ Oral health behaviours include proper brushing, use of fluoride toothpaste, flossing, etc. Studies have found that oral health behaviours and oral habits affect dental health, and that proper oral health behaviours among college students are important for overall health and well-being.^3,9^

In China, there are few studies on the associations between self-rated oral health, subjective oral conditions, oral health behaviours and OHRQoL. In addition, there are not many studies that combining oral health, personal behaviour, and OHRQoL.

This study aims to construct a structural equation model (STEM) to explore the complex associations between them, in order to facilitate fundamental guidance and oral health education of college students.

## Materials and Methods

### Ethical Approval

The protocol of this study was approved by the Medical Ethics Committee of Bengbu Medical College, China (project code 2019-062). All procedures performed were in accordance with the ethical standards of the institutional and national research committee and with the 1964 Helsinki declaration and its later amendments or comparable ethical standards. Before data collection, all participants read the consent information, and provided verbal informed consent by clicking “Continue” to take this online survey. The informed consent protocol was approved by the Medical Ethics Committee of Bengbu Medical College, China. The details of the study and the processing of results were explained in detail before the questionnaire was completed to ensure that each participant was informed and understood. Students who did not want to participate in the study could refuse to fill out the questionnaire; all participants did so voluntarily.

### Subjects and Design

We conducted a cross-sectional research survey of college students from 22 comprehensive colleges in Anhui Province, China, between October 2019 and January 2020. A total of 1708 valid electronic questionnaires were returned using the online survey through a random sampling method within each college. The inclusion criterion was a missing answer rate of less than 10% in the questionnaire.

Moreover, the sample size was calculated according to the formula N = Z²P(1-P)/d^2^ (Z = 1.96, P = 60.1%, d = 0.05).^15^ P was calculated using the oral health knowledge rate of 60.1% of the population from previous survey.^22^ The final minimum sample size obtained was 441. We exceeded the minimum required sample size to ensure the credibility of our results.

### Hypotheses

**Fig 1:**
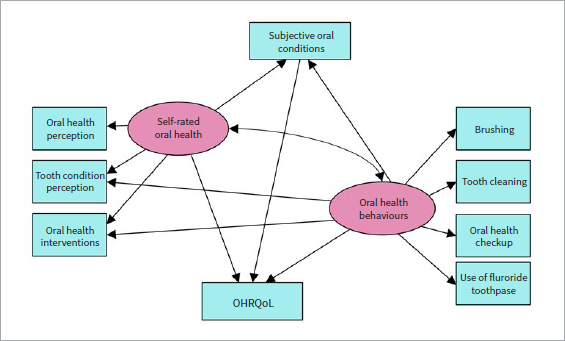
Conceptual model/ structural equation model (STEM) with the hypotheses. Rectangles contain observed variables, ellipses indicate potential variables. OHRQoL: oral health-related quality of life.

The study hypotheses were: self-rated oral health and oral health behaviours have a positive effect on subjective oral conditions and OHRQoL; oral health behaviours are positively related to self-rated oral health; subjective oral conditions are positively related to OHRQoL (Fig 1).

### Survey Instruments

The questionnaire included demographic characteristics, self-rated oral health, subjective oral conditions, oral health behaviours, and OHRQoL.

The demographic characteristics included the age, major, gender, grade and annual household income of the participants.

The first part was self-rated oral health, which consisted of 5 questions. This part was divided into 3 categories. The first category was oral health perception, and the question posed was ‘How would you judge your oral health?’. We asked ‘What is your assessment of your tooth health?’ to determine the second category of tooth condition perception. Answers were given on a 5-point Likert scale to calculate the score. The third category was oral health interventions. It included when and why the last visit to the dentist took place and whether oral health instruction was given. The results were expressed as means ± SD. Higher oral health perception and tooth condition perception scores indicated poorer evaluations. Higher oral health interventions scores indicated better evaluations.

Subjective oral conditions were evaluated using 10 items, including whether the participants have ever had bleeding gums, tooth pain, tooth sensitivity, TMJ pain, dental trauma, sore mouth (stomatitis), difficulty opening the mouth wide, oral mucosal disease, periodontal disease or gingivitis. The score options were ‘0 = never’, ‘1 = occasionally’, ‘2 = frequently’. The results were expressed as the mean ± SD of the scores on the 10 questions. The higher the score, the worse the subjective oral conditions.

Oral health behaviours included 9 questions. Participants filled in the questions according to their actual situation. This part was divided into 4 categories. The first category was brushing, including brush teeth ≥ 2 times a day (B1); brush teeth ≥ 3 min every time; (B2); and change toothbrush every 3 months (B3). The second category was tooth cleaning, including using dental floss to help clean interdental spaces (C1); gargle after meals (C2); clean teeth regularly (C3); brushing method is the ‘horizontal-vibration brushing method’ recommended by the Chinese Stomatological Association (C4). The third category concerned oral health checkups. The question was ‘Do you regularly attend oral health checkups?’. The fourth category was use of fluoride toothpaste, with the corresponding question ‘Do you often use fluoride toothpaste?’. The possible scores were ‘1 = yes’ and ‘0 = no’. The results were expressed using the mean ± SD of the scores in each category. Higher scores indicated better oral health behaviours.

The fourth part concerned OHRQoL. The Chinese version of Oral Health Impact Profile (OHIP)-14 was selected to evaluate OHRQoL. OHIP scores were significantly correlated with life satisfaction.^12^ Response options were ‘4 = often’, ‘3 = fairly often’, ‘2 = occasionally’, ‘1 = hardly ever’, and ‘0 = never’. The higher the OHIP-14 score, the more severely the OHRQoL was influenced by the oral condition.

The overall Cronbach’s α for the questionnaire was α = 0.903. This questionnaire has good internal consistency.

### Statistical Analysis

Frequency, percentage and mean ± SD were used to describe the demographic characteristics of participants. The appropriateness of the scale was checked using the Kaiser-Meyer-Olkin (KMO) measure and the Bartlett spherical test. The KMO coefficient for this questionnaire was 0.927 and the p-value of Bartlett’s test was < 0.001. We used exploratory factor analysis (EFA) to examine the degree of association between questions. A project analysis was carried out to test the degree of differentiation of the questions on a 27% scale.

The associations that existed between self-rated oral health, subjective oral conditions, oral health behaviours, and OHRQoL were analysed by constructing a STEM (structural equation model). First, however, a confirmatory factor analysis (CFA) was performed to assess whether the factor structure chosen based on our study was acceptable or whether it should be modified for these data. When constructing the STEM, each observed variable was imported and calculated as a mean value. We use the Bootstrap method to verify whether there were mediating effects between the variables of interest in the model. The confidence interval (CI) was 95% for the direct and indirect effects of each of the suggested mediating variables beyond zero. The skewness-kurtosis test was employed to check the normality of the observed variables.

The model’s fit was evaluated using the maximum likelihood estimate (MLE), chi-squared/degrees of freedom (χ^2^/df), goodness-of-fit index (GFI), comparative-fit index (CFI), normed-fit index (NFI), incremental-fit index (IFI), adjusted goodness-of-fit index (AGFI), root mean square error of approximation (RMSEA) and other indicators. For CFI, GFI, NFI, IFI and AGFI, a fit index above 0.90 (preferably above 0.95) and an RMSEA less than 0.05 indicated that the model fits well. Regression coefficients were used with a statistical significance level of p < 0.05.

The data were analysed using IBM SPSS Statistics 20.0 and IBM SPSS Amos 24.0 (IBM; Armonk, NY, USA).

## Results

### Demographic Data

Of the 1708 participants, 981 were female (57.4%). Table 1 shows the characteristics of the respondents.

**Table 1:** Characteristics of the participants (n = 1708)

Characteristics		Frequency (n)	Percentage (%)
Age			22.0 ± 4.3 (mean, SD)
Gender	Male	727	42.6
	Female	981	57.4
Major	Stomatology	273	16.0
	Medical non-oral specialty	928	54.3
	Non-medical	507	29.7
Grade	Freshman	451	26.4
	Sophomore	243	14.2
	Junior	469	27.5
	Senior	202	11.8
	Graduate	171	10.0
	Postgraduate	172	10.1
Annual household income	Less than ¥50,000	729	42.7
	¥50, 000 to ¥120,000	705	41.3
	More than ¥120,000	274	16.0

### Descriptive Analysis of Self-rated Oral Health, Subjective Oral Conditions, Oral Health Behaviours and OHRQoL

The overall scores for self-rated oral health ranged from 6–20, with an average score of 11.29 ± 3.26. Oral health perception scored highest on its three subscales (2.95 ± 0.73), followed by oral health interventions (2.15 ± 0.88) and finally tooth condition perception (1.89 ± 1.20). Subjective oral conditions of the survey respondents ranged from 0–19, with a mean of 4.33 ± 3.30. Overall oral health behaviours ranged from 1–9, with a mean of 4.02 ± 2.01. Most students brushed teeth ≥ 2 times a day (77.2%), for ≥ 3 min each time (59.9%), and changed toothbrushes every three months (81.9%). About half of the students rinsed their mouths after meals (41.9%) and brushed with fluoride toothpaste (44.6%). In contrast, fewer students flossed their teeth regularly (22.2%), cleaned their teeth regularly (18.2%), brushed their teeth using the horizontal-vibration brushing method recommended by the Chinese Stomatological Association (39.8%), or had regular oral health checkups (15.6%). OHIP-14 scores range from 0 to 54 with an average of 13.17 ± 11.83.

### Structural Equation Model (STEM)

Table 2 shows the normality results for the latent variables. All variables had an absolute value of skewness < 3 and an absolute value of kurtosis < 8, indicating that the distribution of the sample in the variables is consistent with a normal distribution. The model had a p-value < 0.001, χ^2^ of 54.809 and degrees of freedom of 21, resulting in χ^2^/df = 2.610 (< 3). All indicate that the model is statistically significant. With CFI = 0.979, GFI = 0.993, NFI = 0.967, IFI = 0.979, AGFI = 0.985, RFI = 0.943, PNFI = 0.564, PCFI = 0.571, TLI = 0.964 and RMSEA = 0.031, the model data fit well.

**Table 2:** Descriptive statistics for self-rated oral health, oral health behaviours, subjective oral conditions and OHRQoL

			N (%) (score = 1)	Mean ± SD	Range	Median	Skewness	Kurtosis
Self-rated oral health				11.29 ± 3.26	6.00–20.00	12.00	—	—
	Oral health perception			2.95 ± 0.73	1.00–5.00	3.00	0.261	–1.211
	Tooth condition perception			1.89 ± 1.20	1.00–5.00	1.00	1.234	0.511
	Oral health interventions			2.15 ± 0.88	1.00–4.00	2.33	–0.362	1.519
Oral health behaviours				4.02 ± 2.01	1.00–9.00	4.00	—	—
	Brushing			0.73 ± 0.27	0.00–1.00	0.67	–0.637	–0.538
		B1	1319 (77.2)					
		B2	1024 (59.9)					
		B3	1399 (81.9)					
	Tooth cleaning			0.31 ± 0.31	0.00–1.00	0.25	0.870	–0.179
		C1	380 (22.2)					
		C2	716 (41.9)					
		C3	311 (18.2)					
		C4	681 (39.8)					
	Oral health checkup		268 (15.6)	0.16 ± 0.36	0.00–1.00	0.00	1.887	1.559
	Use of fluroride toothpaste		762 (44.6)	0.45 ± 0.50	0.00–1.00	0.00	0.217	–1.953
Subjective oral conditions				4.33 ± 3.30	0.00–19.00	4.00	1.209	1.982
Oral Health Impact Profile–14				13.17 ± 11.83	0.00–54.00	12.00	0.787	–0.044

### Correlation Analysis Between Variables

**Fig 2:**
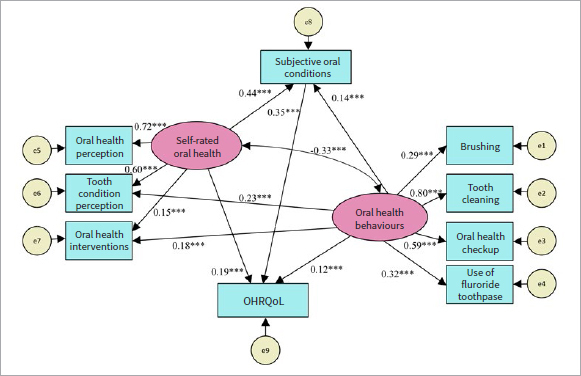
The final structural equation model. Rectangles: observed variables; ellipses: potential variables; circles: residual terms. The values of single-headed arrows represent the standardised coefficients. All pathways are statistically significant (***p < 0.001). OHRQoL: oral health-related quality of life.

Figure 2 shows the standardised estimated parameters of the final structural model. Table 3 shows the results of the correlations between the variables. All paths were statistically significant (p < 0.001). The model showed that: 1. there was a positive correlation between oral health behaviours and self-rated oral health (β = -0.326); 2. oral health behaviours had a positive effect on oral health interventions (β = 0.177); 3. subjective oral conditions had a direct positive effect on OHRQoL (β = 0.346); 4. self-rated oral health had a positive effect on subjective oral conditions (β = 0.442); 5. self-rated oral health had a positive effect on OHRQoL (β = 0.191); 6. oral health behaviours had a negative effect on OHRQoL (β = 0.119), subjective oral conditions (β = 0.143) as well as tooth condition perception (β = 0.595).

**Table 3:** Correlation analysis between variables

Model pathways			Standardised estimate	S.E.	C.R.	p
Subjective oral conditions	←	Oral health behaviours	0.143	0.503	4.385	***
Subjective oral conditions	←	Self-rated oral health	0.442	2.674	4.237	***
Use of fluoride toothpaste	←	Oral health behaviours	0.323	0.070	10.710	***
OHRQoL	←	Subjective oral conditions	0.346	0.093	13.276	***
OHRQoL	←	Oral health behaviours	0.119	1.637	4.009	***
Tooth condition perception	←	Self-rated oral health	0.595	1.244	4.458	***
Oral health interventions	←	Self-rated oral health	0.146	—	—	—
Oral health interventions	←	Oral health behaviours	0.177	0.130	5.594	***
OHRQoL	←	Self-rated oral health	0.191	5.107	3.431	***
Brushing	←	Oral health behaviours	0.285	0.039	9.247	***
Tooth cleaning	←	Oral health behaviours	0.801	0.088	13.112	***
Oral health checkup	←	Oral health behaviours	0.591	—	—	—
Oral health perception	←	Self-rated oral health	0.715	0.962	4.209	***
Tooth condition perception	←	Oral health behaviours	0.231	0.199	6.483	***
Oral health behaviours	↔	Self-rated oral health	-0.326	0.003	-3.582	***

***p < 0.001. S.E.: standard error; C.R.: critical ratio; OHRQoL: oral health-related quality of life.

We used the Bootstrap method to test the mediating effects of the model with a bias correction of 95% CI. The direct and indirect effect results are shown in Table 4. The results showed that oral health behaviours had a direct positive effect on subjective oral conditions, oral health interventions, and tooth condition perception with values of 0.143, 0.177, and 0.231, respectively. This indicated that oral health behaviours had the greatest effect on tooth condition perception. In addition, self-rated oral health had a direct positive effect on subjective oral conditions, as well as subjective oral conditions on OHRQoL, with effect sizes of 0.442 and 0.346, respectively. Furthermore, oral health behaviours had a direct effect on OHRQoL with an effect value of 0.119 and an indirect effect on OHRQoL with an effect size of 0.05 (95% CI of 0.026-0.077). It was shown that subjective oral conditions had a partial mediating effect in the effect of oral health behaviours on OHRQoL. Moreover, there was a direct positive effect of self-rated oral health on OHRQoL, with an effect size of 0.191 and an indirect effect value of 0.153 (95% CI: 0.121-0.191). It indicated that subjective oral conditions also partially mediated the effect of self-rated oral health on OHRQoL.

**Table 4:** Bootstrap analysis of mediating effect significance test for the model

Model pathways			Standardised direct effects	Bias-corrected 95%CI		Standardised indirect effects	Bias-corrected 95%CI	
				LLCI	ULCI		LLCI	ULCI
Oral health behaviors	→	Subjective oral conditions	0.143***	0.075	0.212	—	—	—
Oral health behaviors	→	Oral health interventions	0.177***	0.117	0.238	—	—	—
Oral health behaviors	→	OHRQoL	0.119**	0.056	0.182	0.050***	0.026	0.077
Oral health behaviors	→	Tooth condition perception	0.231***	0.159	0.309	—	—	—
Self-rated oral health	→	Subjective oral conditions	0.442***	0.373	0.510	—	—	—
Self-rated oral health	→	OHRQoL	0.191***	0.118	0.267	0.153***	0.121	0.191
Subjective oral conditions	→	OHRQoL	0.346***	0.288	0.402	—	—	—

All of the direct effects were statistically significant (p < 0.01); **p < 0.01; ***p < 0.001; 95%CI: 95% confidence interval; OHRQoL: oral health-related quality of life.

## Discussion

This study analysed the potential associations between self-rated oral health, subjective oral conditions, oral health behaviours, and OHRQoL among college students by constructing a STEM. The results showed that there were influential associations between the variables. To our knowledge, there are few such studies involving college students in Anhui Province. Our study aimed to explore behaviours and self-perceptions of oral health among college students in advance to improve OHRQoL and further improve health conditions.

A negative correlation was found between worse oral health behaviours and the students’ self-rated oral health. According to the questionnaire scoring design, correct oral health behaviours and good self-rated oral health positively influence each other. Correct oral health behaviours are important determinants of oral health.^5^ Similarly, when people become aware of a problem with their oral conditions, it may lead to changes in health behaviours, such as improving brushing patterns.^23^ Meanwhile, when poor oral health behaviours are identified, they can be improved in order to enhance oral health.^20^

Not surprisingly, the results showed that the better the oral health behaviours, the more oral health interventions were implemented by the students. This is similar to the results of a previous study.^19^ Oral interventions included seeing a dentist and receiving education about oral health. Many studies have found that oral health education can significantly improve oral health behaviours and thus improve oral health.^10,17^ College students are in a transitional stage of health perception and behavioural formation,^12^ and it is important to provide the necessary oral health interventions to make them aware of the importance of oral health.

The present study found that when subjective oral conditions are worse, college students report poorer OHRQoL. This is supported by a study which found statistically significant effects of oral diseases such as oral pain and dental caries on OHRQoL.^26^ When people suffer from toothache, it has a negative impact on their daily work and quality of life.^21^ Another study found that subjective health condition assessments more accurately reflect a patient’s quality of life than do clinical assessments.^25^ College students need to have early oral health check-ups and treatment to avoid negative impacts on their quality of life.

Our results indicated that the better the self-rated oral health status of college students was, the better their subjective oral conditions and OHRQoL were. Other authors found that symptoms of oral diseases such as toothache play an important role in oral health self-assessment.^1^ A Japanese study showed that self-rated oral health had correlated positively with subjective chewing ability and OHRQoL.^26^ Perception of oral health is closely related to awareness of oral disease. In other words, subjective oral conditions also influence college students’ perceptions of their oral health.^8^ When college students have satisfactory oral health, their social interactions with peers will improve, as will their self-confidence, which is important for the well-being and quality of life of today’s youth.^22^

Counterintuitively, we found that when college students had better oral health behaviours, their OHRQoL was sometimes poorer, along with worse subjective oral conditions and poorer tooth condition perception. Other authors found that oral health behaviours in adults have a significant impact on symptoms such as dental caries or tooth loss, thus further influencing OHRQoL.^2^ Our finding can be explained by the fact that dental students made up a small percentage (14.8%) of the population in our survey, and the rest of the college students may not have learned correct brushing or oral cleaning methods. The latter are overly satisfied with their oral health behaviours, but the actual subjective oral and tooth conditions were not satisfactory.

Notably, subjective oral conditions play a partial mediating role in both the effect of oral health behaviours on OHRQoL and the effect of self-rated oral health on OHRQoL. This is similar to previous study.^26^ It indicates that the impact of subjective oral conditions on the quality of life of college students should no be ignored. Thus, it can be assumed that the subjective oral conditions are a prerequisite for OHRQoL. In addition, oral health behaviours had the greatest impact on tooth condition perception. Improving oral health behaviours may be the most effective point of intervention for changing the tooth condition of college students.

A limitation of the present study is that it was conducted only in colleges in Anhui Province; future research will examine the oral health of college students nationwide in depth.

## Conclusion

This study showed that college students in eastern China had positive self-rated oral health and did well on some oral health behaviours, but their subjective oral conditions were not satisfactory. There were influential associations between college students’ self-rated oral health, subjective oral conditions, oral health behaviours, and OHRQoL. In addition, subjective oral conditions play a partially mediating role in both the effect of oral health behaviours on OHRQoL and the effect of self-rated oral health on OHRQoL. Exploiting the association between them can be a guide to fundamentally improve the oral health of college students.
